# Comparison of serological assays to titrate Hantaan and Seoul hantavirus-specific antibodies

**DOI:** 10.1186/s12985-017-0799-0

**Published:** 2017-07-18

**Authors:** Weihong Li, Shouchun Cao, Quanfu Zhang, Jiandong Li, Shuo Zhang, Wei Wu, Jing Qu, Chuan Li, Mifang Liang, Dexin Li

**Affiliations:** 1 Key Laboratory for Medical Virology, National Health and Family Planning Commission of the People’s Republic of China; Laboratory for Viral Hemorrhagic Fever, National Institute for Viral Disease Control and Prevention, China CDC, Beijing, 102206 People’s Republic of China; 2 Institute for Infectious Disease and Endemic Disease Control, Beijing CDC, Beijing, 100013 People’s Republic of China; 3 National Institutes for Food and Drug Control, Beijing, 100050 People’s Republic of China

**Keywords:** Hantaan, Seoul, Serotyping, Plaque reduction neutralization test, Microneutralization test, Pseudoparticle neutralization test, Immunofluorescence assay, Glycoproteins

## Abstract

**Background:**

Hantaan and Seoul viruses, in the Hantavirus genus, are known to cause hemorrhagic fever with renal syndrome (HFRS). The plaque reduction neutralization test (PRNT), as conventional neutralization test for hantaviruses, is laborious and time-consuming. Alternatives to PRNT for hantaviruses are required.

**Methods:**

In this study, the methods for Hantaan and Seoul viruses serological typing including microneutralization test (MNT), pseudoparticle neutralization test (PPNT) and immunofluorescence assay based on viral glycoproteins (IFA-GP) were developed and compared with PRNT using a panel of 74 sera including 44 convalescent sera of laboratory confirmed HFRS patients and 30 patients sera of non-hantavirus infection. Antibody titres and serotyping obtained with different methods above were analyzed by paired-t, linear correlation, McNemar χ^2^ and Kappa agreement tests.

**Results:**

Antibody titres obtained with MNT_50_, PPNT_50_ and IFA-GP were significantly correlated with that obtained with PRNT_50_ (*p* < 0.001). GMT determined by PPNT_50_ was statistically higher than that determined by PRNT_50_ (*p* < 0.001), while GMT determined by MNT_50_ and IFA-GP were equal with (*p* > 0.05) and less than (*p* < 0.001) that obtained with PRNT_50_ respectively. Serotyping obtained with MNT_50_ and PRNT_50_, PPNT_50_ and PRNT_50_ were highly consistent (*p* < 0.001), whereas that obtained with IFA-GP and PRNT_50_ were moderately consistent (*p* < 0.001). There were no significant differences for serotyping between PRNT_50_ and MNT_50_, as well as PRNT_50_ and PPNT_50_ (*p* > 0.05). IFA-GP was less sensitive than PRNT_50_ and MNT_50_ for serotyping of hantaviruses infection (*p* < 0.05). However, for 79.5% (35/44) samples, serotyping determined by IFA-GP and PRNT_50_ were consistent.

**Conclusions:**

MNT_50_ and PPNT_50_ both can be used as simple and rapid alternatives to PRNT_50_, and MNT_50_ is more specific while PPNT_50_ is more sensitive than other assays for neutralizing antibody determination. So far, this work has been the most comprehensive comparison of alternatives to PRNT.

## Background

Hantaviruses belong to the *Hantavirus* genus in the *Bunyaviridae* family [1]. Hantaviruses are enveloped, negative-stranded RNA viruses containing three single-stranded RNA genome segments designated as small (S), medium (M) and large (L); they encode nucleocapsid protein (N), envelope glycoproteins (Gn and Gc) and RNA-dependent RNA polymerase, respectively [2, 3]. Among the viral proteins, nucleocapsid protein possesses an immunodominant antigen, and the antigenicitiy of N protein is conserved compared with that of envelope glycoproteins [4, 5]. Gn and Gc form oligomers on the surface of the virion and are the targets of neutralizing antibodies [6–8].

Hantavirus causes two human diseases: hemorrhagic fever with renal syndrome (HFRS) in Eurasia and hantavirus pulmonary syndrome (HPS) in the Americas. At least four hantaviruses cause HFRS: Hantaan, Seoul, Puumala, and Dobrava viruses caused most of HFRS cases in Eurasia [9, 10]. Hantaan virus (HTNV) and Seoul virus (SEOV) are major causative agents of HFRS in China [11], During the last decade, about 10,000 cases of HFRS were registered annually in China [12]. In general, hantaviruses are host-restricted that Hantaan virus isolates are carried by *Apodemus* and Seoul virus isolates by *Rattus* [1].

The plaque reduction neutralization test (PRNT), is laborious and time-consuming (takes about 2 ~ 3 weeks), and is unsuitable for high-throughput testing [13–15]. Therefore, alternative methods to PRNT are needed.

Microneutralization test (MNT) has been developed for viruses such as influenza virus, Puumala virus, etc. [16–22]. By using 96-well microplates in combination with enzyme immunoassay, MNT is simple, rapid, and adaptable to high-throughput formats.

Pseudotyped reporter viruses containing the envelope glycoprotein of one virus and the core and genome of vector virus such as vesicular stomatitis virus (VSV), murine leukemia virus (MuLV) or lentivirus have been developed for many other viruses [23–27]. Since pseudoparticle is unable to produce infectious progeny viruses unless the envelope proteins are provided in *trans,* pseudoparticle neutralization test (PPNT) is a safe alternative to neutralization test using live viruses. Pseudoparticles bearing glycoproteins of hantaviruses have also been developed and used in PPNT [28–33] for hantaviruses.

Here, we compared the MNT and PPNT data with those obtained with PRNT using 44 convalescent sera from laboratory confirmed patients of HFRS and 30 sera negative for hantavirus infection. Moreover, the effective expressions of glycoproteins of HTNV and SEOV in 293T cells enable us to develop a method of immunofluorescence assay based on viral glycoproteins (IFA-GP) to detect antibody titres against recombinant glycoproteins of the two viruses. The IFA-GP titres may correlate with the neutralizing antibody titres obtained by PRNT, thus IFA-GP has the possibility to be a simpler alternative to PRNT. Here, results obtained with IFA-GP were also compared with that obtained with PRNT using the same panel of sera mentioned above.

## Methods

### Cells, viruses, antibody

Vero-E6 cells (ATCC, C1008 CRL1586) were propagated in growth medium (Eagle’s MEM supplemented with 10% heat-inactivated fetal bovine serum [FBS], 2 mM L-glutamine, 100 U/ml Penicillin, 100 μg/ml Streptomycin, and 1.5 g/L Sodium Bicarbonate solution). HEK 293T human embryo kidney cells (ATCC, CRL11268) and Huh-7.5 human hepatocellular carcinoma cells [34] were propagated in Dulbecco’s Modified Eagle Medium [DMEM] containing 10% heat-inactivated FBS, 100 U/ml Penicillin, 100 μg/ml Streptomycin.

HTNV strain 84FLi and SEOV strain L99 maintained in our laboratory were propagated on Vero E6 cells.

The mouse monoclonal antibodies (mAbs) L13F3 directed against N protein of SEOV and HTNV were generated in our laboratory [35]. Mouse mAbs 8B6 directed against hantavirus Gn glycoprotein [6] and human recombinant mAbs Y5 directed against hantavirus Gc glycoprotein [36] were stored in our laboratory.

### Serum samples

A panel of 74 human sera was used in this study, including 44 convalescent sera from laboratory confirmed patients of HFRS in China, 15 sera from healthy individuals and 15 sera from patients of dengue fever or severe fever with thrombocytopenia syndrome which were negative for hantavirus infection. Sera from patients of HFRS were tested by recombinant nucleocapsid proteins based IgM capture ELISA [37] or IgG ELISA [38] for HTNV and SEOV. Hantavirus infection was confirmed by hantavirus IgM positive or IgG titre ≥4-fold increase between acute-phase and convalescent-phase sera. Real-time RT-PCR developed in our laboratory by Pang et al. [39] was used to differentiate infection of HTNV or SEOV. As shown in Table 1, all 44 HFRS patients were IgM antibodies against hantavirus positive in acute phase sera. 33 of the 44 acute phase sera were tested using real-time RT-PCR, only 5 samples were confirmed to be HTNV or SEOV infection.

**Table 1:** PRNT, MNT, PPNT, IFA-virus and IFA-GP titres of convalescent sera from patients of HFRS

Sero-type^a^	Patient ID	Diagnosis of HFRS^b^				Reciprocal PRNT titre^c^				Reciprocal MNT titre^c^				Reciprocal PPNT titre^c^						Reciprocal IFA titre^c^			
		Acute serum			Convalescent serum	IC50		IC80		IC50		IC80		IC50			IC80			IFA-virus		IFA-GP	
		IgM	IgG	RNA	IgG	SEOV	HTNV	SEOV	HTNV	SEOV	HTNV	SEOV	HTNV	SEOV	HTNV	VSV	SEOV	HTNV	VSV	SEOV	HTNV	SEOV	HTNV
HTN	SD11	+	+	NT	+	20	80	<20	20	20	80	<20	20	80	320	<20	20	80	<20	80	320	20	80
	JX46	+	+	−	+	<20	80	<20	20	<20	80	<20	80	80	320	<20	20	80	<20	80	80	80	80
	SD15	+	+	NT	+	<20	80	<20	20	<20	80	<20	80	80	320	<20	20	80	<20	80	80	20	80
	LN264	+	−	NT	+	<20	80	<20	20	<20	80	<20	20	20	320	<20	20	80	<20	80	80	<20	20
	JX11	+	+	−	+	20	80	<20	80	<20	80	<20	80	20	320	20	<20	80	<20	80	80	20	80
	JX16	+	−	−	+	20	320	<20	80	20	320	<20	80	80	1280	<20	20	320	<20	80	80	20	80
	JX53	+	+	−	+	20	320	<20	80	20	320	20	80	80	320	<20	20	80	<20	20	20	20	80
	JX39	+	+	−	+	20	320	<20	80	<20	320	<20	80	20	1280	<20	20	320	<20	80	80	20	80
	JX36	+	+	−	+	<20	320	<20	80	<20	320	<20	80	20	320	20	20	80	<20	80	320	20	80
	SD7	+	+	−	+	<20	320	<20	80	<20	320	<20	80	320	320	<20	80	80	<20	<20	80	80	80
	SD14	+	+	−	+	<20	320	<20	80	<20	320	<20	80	80	1280	<20	20	320	<20	<20	80	20	80
	SD39	+	+	−	+	<20	320	<20	80	<20	80	<20	80	80	320	<20	20	80	<20	80	80	20	80
	SD21	+	+	H+	+	20	320	20	80	<20	320	<20	80	320	1280	<20	80	320	<20	320	320	80	80
	SD31	+	+	−	+	20	320	<20	80	20	320	<20	80	320	5120	<20	80	320	<20	1280	1280	80	320
	SD38	+	+	−	+	<20	320	<20	80	<20	320	<20	80	80	1280	<20	20	320	<20	80	80	20	80
	JX12	+	+	−	+	<20	320	<20	80	<20	320	<20	80	80	1280	<20	20	320	<20	20	20	20	80
	JX60	+	+	NT	+	20	1280	<20	320	20	1280	<20	320	80	5120	<20	20	1280	<20	320	320	80	320
	JX35	+	+	−	+	<20	1280	<20	320	20	1280	<20	320	320	1280	<20	80	320	<20	80	80	20	80
	SD27	+	+	−	+	20	1280	20	320	20	5120	20	1280	80	5120	<20	20	1280	<20	80	320	80	320
	SD25	+	+	−	+	80	1280	20	320	80	1280	20	320	320	1280	<20	80	320	<20	320	320	80	320
	SD33	+	−	−	+	80	1280	20	320	20	1280	<20	320	320	1280	<20	80	320	<20	320	320	80	320
	SD36	+	+	−	+	<20	1280	<20	320	20	1280	<20	320	320	1280	<20	80	320	<20	80	80	80	80
	SD26	+	+	−	+	<20	1280	<20	320	<20	1280	<20	320	80	1280	<20	20	320	<20	20	80	20	80
	JX3	+	+	−	+	20	1280	<20	320	<20	1280	<20	320	80	1280	<20	20	320	<20	320	320	80	320
	SD29	+	+	H+	+	80	1280	20	320	20	1280	20	320	320	5120	20	80	1280	<20	80	320	20	80
	SD20	+	+	−	+	320	1280	80	320	320	1280	80	320	1280	5120	<20	320	1280	<20	80	80	320	320
	LN34	+	+	NT	+	1280	20,480	320	5120	1280	20,480	320	5120	5120	20,480	20	1280	5120	<20	320	320	5120	5120
SEO	LN216	+	+	NT	+	80	20	20	<20	320	20	80	<20	320	80	<20	80	20	<20	80	80	80	20
	LN28	+	−	NT	+	80	20	20	<20	80	20	20	20	80	<20	<20	20	<20	<20	320	80	80	<20
	LN266	+	+	NT	+	80	20	20	<20	320	<20	80	<20	320	80	<20	80	20	<20	80	80	80	20
	LN265	+	+	−	+	80	20	20	20	80	20	80	20	320	20	<20	80	<20	<20	80	80	80	<20
	LN263	+	+	−	+	80	20	20	20	320	<20	80	<20	320	80	<20	80	20	<20	80	80	80	<20
	LN262	+	+	S+	+	80	20	20	20	320	<20	80	<20	1280	<20	<20	320	<20	<20	320	80	320	20
	JX1	+	−	−	+	320	20	80	20	320	20	80	<20	1280	80	20	80	20	<20	320	320	80	20
	SD4	+	+	−	+	320	80	80	20	320	20	320	<20	1280	20	20	320	<20	<20	80	80	80	<20
	SD2	+	+	−	+	320	20	80	<20	320	<20	80	<20	320	<20	20	80	<20	<20	320	320	80	<20
	JX41	+	+	NT	+	320	<20	80	<20	1280	<20	320	<20	320	80	<20	80	20	<20	80	20	80	20
	SD30	+	+	−	+	320	<20	80	<20	320	<20	80	<20	1280	20	20	320	<20	<20	80	20	80	20
	SD1	+	+	S+	+	320	<20	80	<20	320	20	320	20	1280	20	<20	320	<20	<20	320	320	320	80
	LN27	+	+	NT	+	1280	20	320	<20	1280	<20	320	<20	1280	<20	<20	320	<20	<20	1280	80	320	20
	LN249	+	+	S+	+	1280	320	320	80	1280	80	1280	20	5120	80	<20	1280	20	<20	320	320	1280	320
	SD37	+	+	−	+	5120	320	5120	80	20,480	1280	5120	320	20,480	320	20	5120	80	<20	80	80	1280	320
Unknown	JX22	+	+	NT	+	20	20	<20	<20	<20	20	<20	20	80	80	<20	20	20	<20	80	80	20	20
	SD40	+	+	−	+	320	320	80	80	320	320	80	80	1280	1280	20	320	320	<20	80	80	80	80

^a^Serotype of infection was determined to be HTN (Hantaan), SEO (Seoul) or Unknown by whether antibody level against the homotypic virus was ≥4-fold higher than that of the heterotypic virus

^b^HFRS patients were confirmed by using recombinant nucleocapside proteins based IgM capture ELISA [37] or IgG ELISA [38] for Hantaan virus (HTNV) and Seoul virus (SEOV), and viral RNA was detected by real-time RT-PCR [39] to confirm genotype of infection. +, Positive result. −, Negative result. NT, Not tested. H+, HTNV RNA positive. S+, SEOV RNA positive

^c^Convalescent sera from laboratory confirmed patients of hemorrhagic fever with renal syndrome (HFRS) were titrated against HTNV strain 84FLi and SEOV strain L99 by plaque reduction neutralization test (PRNT), microneutralization test (MNT), pseudoparticle neutralization test (PPNT), immunofluorescence assay based on virus-infected cells(IFA-virus) and immunofluorescence assay based on viral glycoproteins (IFA-GP). PRNT, MNT, PPNT and IFA titres were expressed as the reciprocal of the highest dilution of serum resulting in specific reduction of the number of virus plaques or absorbance value or luciferase activity or fluorescence by 50% (IC50) and 80% (IC80), respectively. IC50, 50% inhibition concentration. IC80, 80% inhibition concentration

### Plaque reduction neutralization test

Methods used for PRNT were essentially the same as previously described [40]. The viral stocks of 84FLi and L99 were titred three times by semi-log10 dilutions by plaque forming assay. The same virus stocks were used in MNT. Then serial 4-fold dilutions of human sera (starting at 1:20) were incubated with 100 PFU of 84FLi or L99 at the final volume of 100 μl at 4 °C overnight. The mixtures were added to confluent monolayers of Vero-E6 cells grown in 6-well plates in duplicates and incubated at 37 °C in a humidified 5% CO_2_ atmosphere for 2 h. Then 4 ml of growth medium containing 10% FBS, 0.6% agarose and 1% DMSO (Sigma) was added to each well. After an incubation at 37 °C in a humidified 5% CO_2_ atmosphere for 7 days (SEOV) and 9 days (HTNV), the plaques were visualized by adding a second overlay identical to the first but containing 2% FBS, 3% neutral red and without DMSO (4 ml per well). The plates were incubated at 37 °C, 5% CO_2_ atmosphere and plaques were observed for 2 ~ 4 days. The 50% inhibition concentration (IC50) and 80% inhibition concentration (IC80) were determined as the reciprocal of the highest dilution of serum resulting in 50% (PRNT_50_) and 80% (PRNT_80_) reduction of plaques as compared to the virus control, respectively.

### Microneutralization test

Microneutralization tests for HTNV and SEOV were developed according to MNT for influenza virus with minor modifications [21]. Direct ELISA and indirect ELISA using mAbs L13F3 were compared by evaluating their P/N ratios.

The viral stocks of 84FLi and L99 were titred at semi-log10 dilutions and the TCID_50_ were calculated by the method of Reed-Munch. Serial 4-fold diluted human sera were mixed with 100 TCID_50_ of 84FLi or L99 in duplicate in microplates and incubated at 4 °C overnight. The confluent monolayers of Vero-E6 cells were trypsinized and 4 × 10^4^ cells in 0.1 ml volume complete EMEM containing 10% FBS, were added to each well containing the virus-serum mixtures in microplates, and incubated at 37 °C in a humidified 5% CO_2_ atmosphere for 7 days. Then the supernatant was removed and monolayers were fixed with precooled 80% (*v*/v) solution of acetone-PBS for 20 min at 4 °C. Subsequently, the acetone-PBS was removed. Then HRP-conjugated mAbs L13F3 diluted with PBS containing 10% FBS and 0.05% Tween 20 were added to the acetone fixed monolayers. After an incubation of 45 min at 37 °C, the plates were washed and TMB was added to develop color. The absorbance values at 450 nm (OD450) were observed by Varioskan LUX multimode microplate reader (Thermo, USA). The IC50 and IC80 were determined as the reciprocal of the highest dilution of serum resulting in 50% (MNT_50_) and 80% (MNT_80_) reduction of OD450 values as compared to the virus control. Positive, negative controls and virus titrations were included in each assay.

### Expression of viral glycoproteins and IFA-GP

RNA was extracted from culture supernatants of 84FLi and L99 infected Vero-E6 cells using QIAamp Viral RNA kit (Qiagen, Germany), and cDNA was prepared by reverse transcription using random primers. To generate glycoprotein expression plasmids, full-length M genes were amplified and cloned into the pCAGGS-MCS vector to generate pCHTNM and pCSEOM.

The HTNV and SEOV glycoproteins were expressed in 293T cells and identified by IFA. Briefly, 293T cells grown on 100-mm dish were transfected with plasmids of pCHTNM, pCSEOM and pCAGGS separately. After 48 h, the 293T cells were trypsinized, fixed with acetone for 20 min, and treated with mouse mAbs 8B6 against hantavirus Gn glycoprotein and human recombinant mAbs Y5 against hantavirus Gc glycoprotein, serum from laboratory confirmed patient of HFRS, and serum from healthy human for 45 min at 37 °C. After washing with PBS, fluorescein isothiocyanate (FITC)-conjugated goat anti-mouse IgG or goat anti-human IgG diluted in PBS containing 5% skim milk, were added. After 45 min incubation at 37 °C, cells were washed with PBS and examined with a fluorescence microscope.

The immunofluorescence assay using recombinant glycoproteins of HTNV and SEOV as antigen to detect anti-glycoprotein antibodies described in this part was designated as IFA-GP.

### Production of lentiviral pseudotyped particles bearing glycoproteins of HTNV and SEOV and pseudoparticle neutralization test

293T cells were seeded in 100-mm diameter dishes 8 ~ 18 h prior to transfection. 12 μg of the glycoprotein expression plasmids of HTNV or SEOV were co-transfected with 12 μg of a lentivirus-based luciferase reporter construct pNL4–3.Luc.R-E- [41] with X-treme GENE HP DNA Transfection Reagent (Roche, Switzerland). Vesicular stomatitis virus glycoprotein envelope (VSV-G)-pseudotyped lentiviral particles were made and used as control to evaluate non-specific neutralization caused by inhibition materials to transduction of lentivirals. Transfection reagent was added to plasmids diluted in opti-MEM (Gibco, USA), and incubated at room temperature for 15 to 20 min, then added dropwise to 293T cells and incubated at 37 °C for 8 h. Then the medium was replaced with fresh medium containing 2% FBS. Culture medium containing pseudoparticles of HTNV (HTNVpp) and SEOV (SEOVpp) were collected at 24 and 48 h after transfection and clarified by centrifugation at 900 x g for 10 min. To make virus stocks used in PPNT, 1 volume of lenti-X concentrator (Clontech, USA) was combined with 3 volumes of the clarified supernatant, the mixture were incubated at 4 °C overnight, and then centrifuged at 1500 x g for 45 min at 4 °C. After centrifugation, the supernatant was removed carefully, the pellet was suspended in DMEM containing 10% FBS. Single use aliquots were frozen with liquid nitrogen and stored at −80 °C.

Pseudoparticle neutralization tests for HTNV and SEOV were carried out as previously described for SFTSV with some modifications [27]. HTNVpp and SEOVpp stocks were titrated. Then, serial 4-fold dilutions of heat inactivated human sera were mixed with 3000 relative luciferase unit (RLU) of HTNVpp or SEOVpp in duplicate in 96-well plate for 1 h at 37 °C in a total volume of 100 μl. Then the virus-sera mixtures were added to Huh-7.5 cells plated in black 96-well plates 24 h before. After 48 h, the luciferase activities in cell lysates were measured with One-Glo Luciferase Assay System and Varioskan LUX multimode microplate reader (Thermo, USA). The IC50 and IC80 were determined as the reciprocal of the highest dilution resulting in 50% (PPNT_50_) and 80% (PPNT_80_) reduction of luminescence values as compared to positive control, respectively.

### Immunofluorescence assay using virus-infected cells (IFA-virus)

Vero-E6 cells infected with 84FLi or L99 were maintained in complete EMEM containing 2% heat-inactivated FBS and incubated for 7 days at 37 °C. Then cells were trypsinized and spotted onto 8-well slides, air dried and fixed with acetone. These slides were incubated with 4-fold serial dilutions of human sera (starting at 1:20 dilution), and probed with FITC-conjugated goat anti-human IgG. IFA titres were expressed as the reciprocal of the highest dilution of serum resulting in specific fluorescence of hantaviruses.

### Comparison of antibody titres and serotyping obtained by PRNT, MNT, PPNT, IFA-GP

Serotype of infection was determined by whether antibody level against the homotypic virus was ≥4-fold higher than that of the heterotypic virus. Antibody titres and serotyping results obtained by methods above were compared using statistical analysis.

### Statistics

The difference of geometric mean titres (GMTs) were evaluated by paired-t test and the correlations between methods were analyzed by linear correlation test using GraphPad Prism 5 and IBM SPSS statistics 19.

Results of serotyping between methods were compared by McNemar χ^2^ tests on cross-table (for categorical variable) and Kappa agreement tests using IBM SPSS statistics 19.

A *P* value less than 0.05 was considered statistically significant.

## Results

### Virus titration by plaque forming assay

**Fig. 1:**
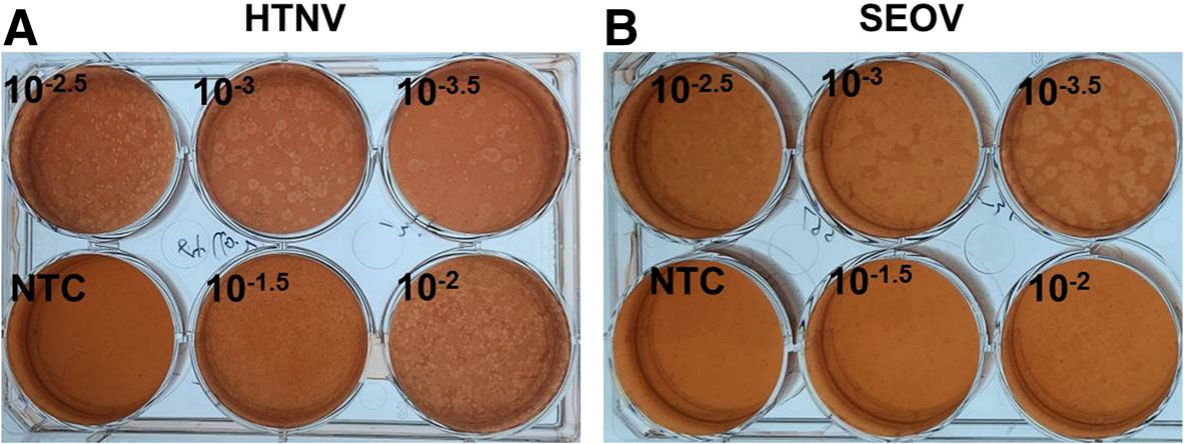
Virus titration by plaque forming assay. **a** Hantaan virus strain 84FLi; **b**, Seoul virus strain L99

Figure 1 shows the results of plaque forming assay. The titre (PFU/ml) was 1.6 × 10^6.0^ for 84FLi and 5.1 × 10^6.0^ for L99.

#### Microneutralization test

To choose a better detection method in MNT, direct ELISA and indirect ELISA in MNT were compared.

**Fig. 2:**
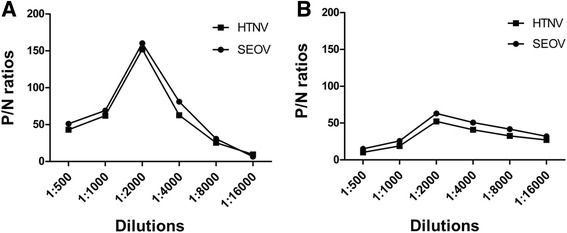
Comparison of detection methods in MNT. **a** direct ELISA; **b** indirect ELISA. P/N ratios, positive/negative ratios

1:2000 diluted mAb L13F3 achieved the best P/N ratio for both direct ELISA and indirect ELISA (Fig. 2), and direct ELISA produced higher P/N ratio than indirect ELISA (Fig. 2). Since direct ELISA is simpler and more time-saving than indirect ELISA, it was used in MNT.

**Fig. 3:**
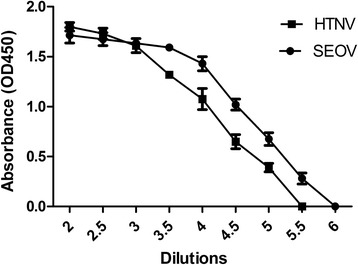
Microtitration of Hantaan virus strain 84FLi and Seoul virus strain L99 used in MNT. Data are means of three experiments. The error bars indicate standard deviations (SD)

Viral stocks were titrated using direct ELISA. The titres of both virus stocks were 2.0 × 10^6.0^ TCID_50_/ml for 84FLi and 6.3 × 10^6.0^ TCID_50_/ml for L99 (Fig. 3).

### IFA detection of HTNV and SEOV glycoproteins expressed in 293T cells

**Fig. 4:**
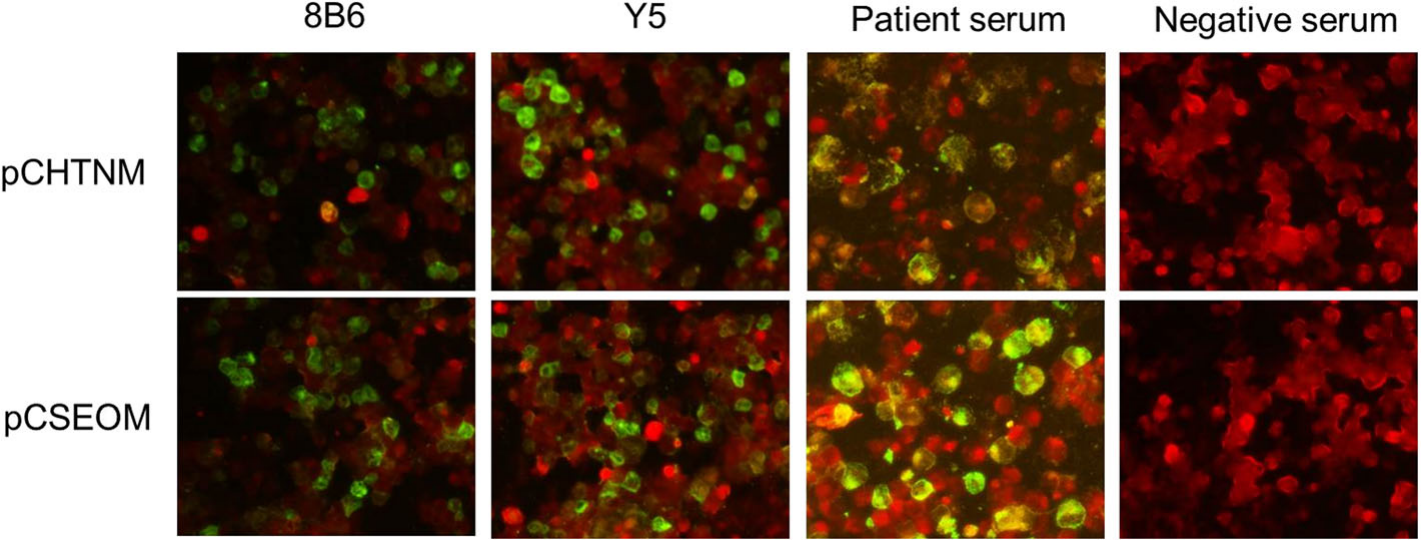
Immunofluorescence detections of glycoproteins of Hantaan and Seoul viruses expressed in 293T cells. pCHTNM, Recombinant expressing plasmid for glycoprotein of Hantaan virus strain 84FLi. pCSEOM, Recombinant expressing plasmid for glycoprotein of Seoul virus strain L99

IFA assays showed that both Gn and Gc glycoproteins of HTNV and SEOV were effectively expressed in transiently transfected 293T cells. Thus, the recombinant viral glycoproteins can be used as antigen to detect hantavirus-specific antibodies in human sera by IFA (Fig. 4).

### Titration of pseudoparticles of HTNV and SEOV

**Fig. 5:**
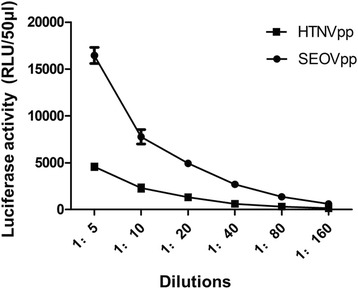
Titration of HTNVpp and SEOVpp on Huh-7.5 cells. Data are means of three experiments. The error bars indicate standard deviations (SD)

HTNVpp and SEOVpp were titrated on Huh-7.5 cells. The infectious titre (RLU/ml) was 2.0 × 10^5^ for HTNVpp and 8.0 × 10^5^ for SEOVpp (Fig. 5). A comparable amount of HTNVpp or SEOVpp giving an RLU of 3000 was incubated with various dilutions of sera was used in PPNT.

### Comparisons of antibody titres obtained by PRNT, MNT, PPNT, IFA-GP and IFA-virus

All 74 human sera were assayed by PRNT, MNT, PPNT, IFA-GP and IFA-virus. There were no non-specific neutralization for all negative sera at 1:20 dilution for PRNT, MNT, IFA-GP and IFA-virus. For PPNT_50_, 46.7% (14/30) of negative sera yielded inhibition of HTNVpp, SEOVpp and VSVpp, no non-specific neutralization found at dilutions ≥1:80 of the sera. The cutoff level for a positive test was set at 1:80 for PPNT_50_.

For 44 convalescent sera of HFRS patients, antibody titres determined by PRNT, MNT, PPNT, IFA-GP and IFA-virus are listed in Table 1.

As shown in Table 1, antibody titres determined by 50% inhibition concentration were relatively higher than that determined by 80% inhibition concentration. PRNT_50_, MNT_50_ and PPNT_50_ were used for the evaluation of these methods.

**Fig. 6:**
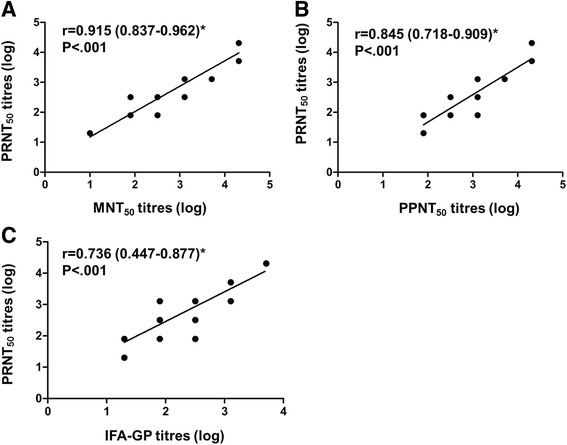
Comparisons of antibody titres. Each dot represents the log10 antibody titre of a serum against Hantaan virus strain 84FLi or Seoul virus strain L99. Total number of sera assayed is 44. The best-fit lines are shown on each graph. On the graphs, r and P indicate the correlation coefficient with 95% confidence interval and the *P* value of significance, respectively. *95% confidence interval of correlation coefficient

To evaluate the correlations of titres obtained with mentioned methods, linear correlation analyses were performed. As shown in Fig. 6a and b, titres obtained with MNT_50_ and PRNT_50_, PPNT_50_ and PRNT_50_, were both highly correlated (*r* > 0.8, *p* < 0.001), reflecting the reliability of these methods. As shown in Fig. 6c, titres determined by IFA-GP and PRNT_50_ were moderately correlated (*r* = 0.736, *p* < 0.001). There were no significant differences among all correlation coefficients as judged by their 95% confidence intervals (Fig. 6).

**Fig. 7:**
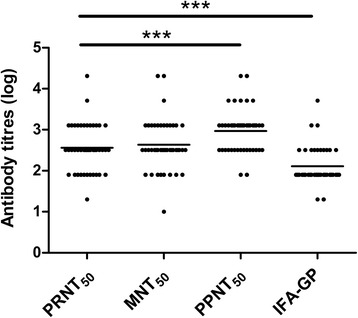
Comparisons of MNT_50_, PPNT_50_, and IFA-GP titres with PRNT_50_ titres. Each dot represents the antibody titres (log) of a serum against Hantaan virus strain 84FLi or Seoul virus strain L99. Total number of sera assayed is 44. Horizontal bars indicate the respective group mean. ***P < .001

When comparing the GMTs obtained with these methods by paired-t test, as shown in Fig. 7, GMT determined by PPNT_50_ was significantly higher than that obtained with PRNT_50_ (*p* < 0.001), while GMT determined by MNT_50_ was statistically equal with that obtained by PRNT_50_ (*p* > 0.05), and GMT determined by IFA-GP was significantly less than that of PRNT_50_ (*p* < 0.001). These results indicate that PPNT_50_ was the most sensitive method for titrating antibody.

To determine the consistency of serotyping obtained with these methods, Kappa agreement tests were performed. Results of serotyping determined by MNT_50_ and PRNT_50_, PPNT_50_ and PRNT_50_ were both highly consistent (kappa = 1.00 or 0.96; *p* < 0.001); whereas that determined by IFA-GP and PRNT_50_ were only moderately consistent (kappa = 0.73, *p* < 0.001).

Finally, no statistically significant differences were observed for serotyping between PRNT_50_ and MNT_50_, as well as PRNT_50_ and PPNT_50_ (*p* > 0.05). It was found that IFA-GP was statistically less sensitive (*p* < 0.05) than PRNT_50_ and MNT_50_ for serotyping of hantaviruses infections. Seven sera, which could be serotyped by PRNT_50_ and MNT_50_, could not be serotyped by IFA-GP.

Overall, MNT_50_ was more specific while PPNT_50_ was more sensitive for titrating neutralizing antibody than other assays. IFA-GP was less sensitive than other neutralization tests for serotyping.

## Discussion

HFRS was caused by HTNV and SEOV in China, no other hantavirus infection of human has been comfirmed. HTNV and SEOV have discriminative antigenicities, and are carried by different rodents. For vaccine development, diagnosis and rodents control, it is necessary to identify the type of hantavirus that cause human disease. Molecular genotyping including conventional PCR and real-time RT-PCR have been developed for Hantavirus to detect viral RNA and genotyping in clinical samples [42, 43]. Because of very low virus load and shortness of viremia of HFRS patients, the nucleic acid test is not sensitive enough to be implemented in clinical diagnosis of HFRS routinely [42, 43]. In this study, 33 acute phase sera of HFRS patients were tested by real-time RT-PCR, only 5 sera have been genotyped. Instead, serological assays to detect IgM and/or IgG antibodies by ELISA or IFA have been broadly used. Chu et al. explored the antigenic relationships among 32 hantavirus isolates, they found most of the SEO-like and HTN-like viruses displayed strong two-way cross-reactivity tested by ELISA [40]. The analysis of mAbs against N protein of HTNV and SEOV showed the most mAbs react with both HTNV and SEOV [5]. As the “gold standard” for Hantavirus serotyping, PRNT has been broadly used. Determination of neutralizing antibody is necessary for doing population based seroepidemiological survey assessing the exposure of population to these viruses or evaluating immune effect of vaccine. PRNT is time-consuming, laborious and need to handle with living virus. Some easy accessing and safe methods for neutralizing antibody detection were established and preliminary evaluated.

As compared with PRNT, MNT is more objective, simpler and time-saving for high throughput detection of virus neutralizing antibodies. The same virus stocks were used in both MNT and PRNT, so neutralizing antibody titres determined by MNT_50_ and PRNT_50_ were highly correlated.

However, the MNT for Hantavirus still need to handle with living viruses. PPNTs for HTNV and SEOV have been developed, which can be completed in 3 ~ 4 days and don't need high biosafety level facility since the pseudotyped virus is unable to produce infectious viruses. In this study, neutralizing antibody titres obtained with PPNT_50_ were significantly higher than that obtained with PRNT_50_. Although one serum (SD7) was determined as infection of HTNV by PRNT_50_ and MNT_50_, it cannot be serotyped by PPNT_50_. For most sera from HFRS patients, the neutralizing antibody determined by PPNT were consistent with that determined by neutralization tests using live viruses.

While IFA-virus can’t differentiate infection of HTNV and SEOV is a common opinion [44], IFA-GP has not been reported for hantaviruses serotyping. Here, IFA-GP was less sensitive than PRNT_50_ and MNT_50_ for serotyping infections of hantaviruses. Seven sera, which could be serotyped by PRNT_50_ and MNT_50_, could not be serotyped by IFA-GP. Some of the envelope glycoprotein antigenic determinants are not involved in virus neutralization [6], so anti-glycoprotein antibody titres may not be fully correlated with neutralizing antibody titres. However, for 79.5% (35/44) samples, serotyping obtained with IFA-GP and PRNT_50_ were consistent. In view of the simplicity of IFA-GP, it is meaningful for serotyping.

## Conclusion

While PRNT is the standard neutralization test for hantaviruses, MNT_50_ and PPNT_50_ both can be used as simple and rapid alternatives to PRNT_50_ for Hantaan and Seoul viruses. MNT_50_ is more specific while PPNT_50_ is more sensitive than other assays for neutralizing antibody determination. IFA-GP is meaningful for serotyping in view of its simplicity. So far, this work has been the most comprehensive comparison of alternatives to PRNT.

## Abbreviations

DMEM: Dulbecco’s Modified Eagle Medium
ELISA: Enzyme linked immunosorbent assay
FBS: Fetal bovine serum
FITC: Fluorescein isothiocyanate
GMT: Geometric mean titre
HFRS: Hemorrhagic fever with renal syndrome
HRP: Horseradish peroxidase.
HTNV: Hantaan virus
HTNVpp: Pseudoparticles of HTNV
IC50: 50% inhibition concentration
IC80: 80% inhibition concentration
IFA: Immunofluorescence assay
IFA-GP: Immunofluorescence assay based on viral glycoproteins
IFA-virus: Immunofluorescence assay using virus-infected cells
mAbs: Monoclonal antibodies
MNT: Microneutralization test
MuLV: Murine leukemia virus
N: Nucleocapsid protein
OD450: Absorbance value at 450 nm
P/N: Positive/ Negative
PFU: Plaque-forming units
PPNT: Pseudoparticle neutralization test
PRNT: Plaque reduction neutralization test
RLU: Relative luciferase unit
SD: Standard deviation
SEOV: Seoul virus
SEOVpp: Pseudoparticles of SEOV
TCID_50_: Tissue culture infection dose_50_
VSV: Vesicular stomatitis virus
VSV-G: Vesicular stomatitis virus envelope glycoprotein
VSVpp: Pseudoparticles of vesicular stomatitis virus
